# Sustainability of healthcare innovations (SUSHI): long term effects of two implemented surgical care programmes (protocol)

**DOI:** 10.1186/1472-6963-12-423

**Published:** 2012-11-23

**Authors:** Stephanie M C Ament, Freek Gillissen, José M C Maessen, Carmen D Dirksen, Trudy van der Weijden, Maarten F von Meyenfeldt

**Affiliations:** 1 Department of General Practice, CAPHRI, School for Public Health and Primary Care, Maastricht University Medical Centre, P.O. box 616 , 6200, MD, Maastricht, The Netherlands; 2 Department of Oncology, GROW, School for Oncology & Developmental Biology, Maastricht University Medical Centre, P.O. box 5800 , 6202, AZ, Maastricht, The Netherlands; 3 Department of Surgery, Maastricht University Medical Centre, P.O. box 5800 , 6202, AZ, Maastricht, The Netherlands; 4 Department of Clinical Epidemiology and Medical Technology Assessment, KEMTA, Maastricht University Medical Centre, P.O. box 5800 , 6202, AZ, Maastricht, The Netherlands; 5 Department of Patient & Care, Maastricht University Medical Centre, P.O. box 5800 , 6202, AZ, Maastricht, The Netherlands

## Abstract

**Background:**

Two healthcare innovations were successfully implemented using different implementation strategies. First, a Short Stay Programme for breast cancer surgery (MaDO) was implemented in four early adopter hospitals, using a hospital-tailored implementation strategy. Second, the Enhanced Recovery After Surgery (ERAS) programme for colonic surgery was implemented in 33 Dutch hospitals, using a generic breakthrough implementation strategy. Both strategies resulted in a shorter hospital length of stay without a decrease in quality of care. Currently, it is unclear to what extent these innovative programmes and their results have been sustained three to five years following implementation. The aim of the sustainability of healthcare innovations (SUSHI) study is to analyse sustainability and its determinants using two implementation cases.

**Methods:**

This observational study uses a mixed methods approach. The study will be performed in 14 hospitals in the Netherlands, from November 2010. For both implementation cases, the programme aspects and the effects will be evaluated by means of a follow-up measurement in 160 patients who underwent breast cancer surgery and 300 patients who underwent colonic surgery. A policy cost-effectiveness analysis from a societal perspective will be performed prospectively for the Short Stay Programme for breast cancer surgery in 160 patients. To study determinants of sustainability key professionals in the multidisciplinary care processes and implementation change agents will be interviewed using semi-structured interviews.

**Discussion:**

The concept of sustainability is not commonly studied in implementation science. The SUSHI study will provide insight in to what extent the short-term implementation benefits have been maintained and in the determinants of long-term continuation of programme activities.

## Background

Quality improvement strategies are used to change and improve healthcare. However, often healthcare innovations barely get incorporated in every day practice [1-4]. Implementing an innovation is a complex process. Studies show that 30-40% of patients do not receive healthcare according to evidence-based knowledge and 20-25% of the healthcare provided is not needed or potentially harmful [5-7]. There is a high level of agreement between all parties involved in healthcare that the quality of care could be improved. Currently, there is no consensus on how to achieve better care. A diversity of quality improvement strategies to change and improve healthcare routines has been proposed. However, there is not one strategy that is superior in its effect above other strategies in improving healthcare processes [7]. Although magic bullets are lacking among the prevailing implementation strategies, evidence suggests that implementation strategies which are systematically tailored to the actual and perceived barriers and facilitators for change increase the success of implementation [8,9].

Less is known on the long term effectiveness of implementation strategies. Even after successful implementation, practice seems to indicate that there is a tendency to return to old routines after implementation activities have ended. It can be argued that without consolidation of the achieved benefits, implementation strategies are a waste of time and money. Implementation strategies’ ‘good value for money’ is determined by its ability to implement evidence based clinical guidelines into daily routines and by the implementation costs [10]. The implication of discontinued programmes is however not just financial but can also result in less optimal care and service for patients. Moreover, it can also diminish community trust and support for future programmes. Therefore, it is important that achieved benefits of a proven effective intervention are sustained after implementation.

Sustainability is a relatively new term in healthcare research, but has become an issue of growing interest. There is no uniform definition of sustainability in the literature [11]. In its simplest form, it can be seen as ‘holding the gains’ and ‘evolving as required’. Different definitions regarding sustainability are used, for example “maintaining the health benefits [12]”, “continuation of the programme activities within an organisational structure [13]” and “building the capacity of a recipient community [14]” [11,15,16]. Sustainability is generally seen as a dynamic process of continuous improvement. In the current study we use the following definition: “*Sustainability of change exists when a newly implemented innovation continues to deliver the achieved benefits over a longer period of time*, *certainly does not return to the usual processes and becomes* ‘*the way things are done around here*’, *until a better innovation comes along*, *even after the implementation project is no longer actively carried out*[11,17]”

Sustainability is obtaining its position in healthcare research, but existing work on sustainability has mainly been undertaken from a pragmatic perspective and was impoverished by a lack of process information [18]. Several determinants can have a facilitating or impeding effect on sustainability. Important questions that still need to be answered are by what strategies particular healthcare innovations are implemented and sustained (or not) in particular contexts and settings, and whether these strategies can be improved.

Few empirical studies have specifically addressed the implementation and sustainability of innovations. In the current study two Dutch implementation cases are used for research on sustainability and its determinants (Additional file 1 and Additional file 2). A Short Stay Programme (MaDO) was implemented in breast cancer surgery in 4 early adopter hospitals by means of a hospital tailored implementation strategy [19-22], and the Enhanced Recovery After Surgery (ERAS) programme in colonic surgery was implemented in 33 hospitals by means of a generic implementation strategy [23,24]. As a result of these efforts (Table 1), both cases showed improved short-term results in terms of hospital length of stay. However, it is currently unknown to what extent and how these programmes and their results have been sustained.

**Table 1:** Overview implementation cases

	**MaDO**	**ERAS**
**No of participating hospitals**	4	33
**Innovation**	Ultra Short stay programme after breast cancer surgery	ERAS programme in colonic surgery
**Implementation strategy**	Hospital tailored strategy	The Breakthrough Series
**Implementation time span**	2005 – 2007	2006 – 2009
**Measurement periods**	Two measurement periods of six months, with six months of actual implementation in between	Three runs of each one year

### Objectives

In the present study we will assess sustainability of a Short Stay Programme in breast cancer surgery and the Enhanced Recovery After Surgery programme in colonic surgery, three to five years after their implementation.

The research questions are:

1. To what extent have the achieved benefits of the Short Stay Programme in breast cancer surgery and the Enhanced Recovery After Surgery programme in colonic surgery been sustained?

2. What is the current policy cost-effectiveness of the Short Stay Programme in breast cancer surgery?

3. Which are the determinants of sustainability as perceived by the professionals of the Short Stay Programme in breast cancer surgery and the Enhanced Recovery After Surgery programme in colonic surgery?

## Methods

### Design

The present study is an observational study using a mixed methods approach, applied to the two implementation cases. First, by means of a retrospective analysis of patient files we will analyse process and outcomes of care to assess the extent of sustainability. Second, the policy cost-effectiveness will be calculated for the Short Stay Programme based on prospective data from breast cancer surgery patients. The cost-effectiveness analysis will use the same methodology as in the primary implementation study [19]. Third, the determinants of sustainability will be explored by means of in-depth semi-structured interviews with relevant stakeholders and external change agents. This study will be executed between November 2010 and November 2013.

### Ethical approval and informed consent

The Medical Ethical Committee of the University of Maastricht has granted approval, METC 11-4-015.10. The privacy of the included patients is protected, and all data will be coded and processed anonymously. Medical files with explicit patient statements that their medical information should not be used for clinical research will not be included. Breast cancer patients eligible for the second research question in this study will be asked for informed consent for prospective collection of quality of life and cost data.

### Participants

#### Hospitals

Hospitals participating in the breast cancer surgery case are those four hospitals which participated in the primary implementation strategy, covering four main organisational hospital settings in the Netherlands. The uptake of the Short Stay Programme in breast cancer surgery was successful in all four hospitals; after implementation the average proportion of patients treated in short stay was increased with 36%.

Hospitals participating in the colonic surgery case were selected out of the 33 hospitals which participated in the primary implementation strategy. Selection was based on the criterion that the implementation strategy was successfully executed in these hospitals, such that the chance of finding information on sustainability and its determinants is increased. Success was defined as follows: (1) a median hospital length of stay of six days or lower, (2) an overall protocol compliance above seventy percent, and (3) at least forty patients treated according to the ERAS programme. Ten hospitals were selected for the SUSHI study. Nine hospitals fulfilled the criteria and one hospital was selected on top of that, based on being a successful early adopter hospital.

**Research question one**: *To what extent have the achieved benefits of the Short Stay Programme in breast cancer surgery and the Enhanced Recovery After Surgery programme in colonic surgery been sustained*?

### Participants

#### Patients

Data collection will be conducted among the last 160 consecutive patients who had been scheduled for breast cancer surgery in short stay (40 per centre) and the last 300 consecutive patients who underwent colonic surgery (30 per centre), who met the same inclusion criteria as in the primary implementation studies. Patients in the breast cancer surgery case are all patients aged over 18 years, diagnosed with breast cancer, and who underwent surgery. Patients whose physiology at diagnosis impeded participation in the short stay programme as assessed by the breast surgeon, anaesthesiologist and nurse, will be excluded from the study. Patients who could not rely on sufficient informal home care during the first night after surgery and patients with complaints that necessitated postoperative monitoring and were scheduled for at least one overnight stay, will also be excluded. Patients included in the colonic surgery case were all patients aged over 18 undergoing an elective colonic resection above the peritoneal reflection for both malignant and benign diseases. Patients undergoing emergency surgery and requiring an ileo- or colostomy are excluded.

### Variables and measurements

Outcome and process of care will be measured by means of pre-defined indicators to determine the extent of sustainability of both programmes, which will be extracted from patient files. The primary outcome measure in the breast cancer surgery case is the proportion of breast cancer surgery patients treated in day care admission or one overnight stay. Secondary outcome measure is the number of complications. The primary outcome measure in the colonic surgery case is the hospital length of stay (LOS). Secondary outcome measure is Functional Recovery (FR), reached when a patient is tolerating solid foods, is comfortable on oral analgesics only, and mobilised at preoperative level [25]. Baseline patient characteristics and possible reasons for delay in discharge (the gap between FR and LOS) will be recorded (Table 2).

**Table 2:** Outcome indicators and baseline characteristics scored in both cases

** MaDO**	** ERAS**
*Primary outcome*	*Primary outcome*
Treated surgically in day care	Postoperative hospital length of stay
Treated surgically in overnight stay	Day functional recovery was reached
*Baseline characteristics*	*Baseline characteristics*
Patient characteristics	Patients characteristics
Eligible for surgery in short stay	Laparoscopic or open approach
Receiving breast conserving surgery	Different types of operations

The process indicators are shown in Table 3. For both the breast cancer surgery and colonic surgery case, the outcome measure is the adherence to the protocol. Adherence to the protocol will be determined per process indicator and per patient. Reasons for non-adherence to specific guideline elements will be recorded if available.

**Table 3:** Process indicators in both cases

** MaDO**	** ERAS**
Preoperative counselling	Preoperative counselling
	No preoperative bowel preparation
	Preoperative PreOp carbohydrate drink
Not planned for short stay admission despite fulfilling the inclusion criteria for short stay admission	Epidural anaesthesia/analgesia
Being offered home care nursing after breast cancer surgery	Perioperative warming (Bair hugger)
	No abdominal drains placed during surgery
	Nasogastric tube removed after surgery
	Nutritional supplements postoperatively
	Mobilisation > 15 minutes at day 0
	Use of oral fluids >500 ml at day 0
	Mobilisation > 3 hours at day 1
The reasons for discrepancy between fulfilling the inclusion criteria for short stay admission and being scheduled for inpatient admission	Intravenous fluid infusion stopped at day
	Resumption of solid food at day 1
	Removal of epidural analgesia on day 2
	Oral laxatives postoperatively
The reasons for not being treated in short stay despite being scheduled for short stay	
	The reasons for not adhering to these different protocol elements

### Data analysis

Descriptive statistics (proportions, mean, median) will be performed for both outcome data sets. The percentage adherence to the protocol will be calculated per programme element for both cases and an overall protocol adherence score will be calculated per patient. All statistical analyses will be performed using SPSS 18.0 (SPSS Inc. Chicago, Illinois, USA).

**Research question two**: *What is the current policy cost*-*effectiveness of the Short Stay Programme in breast cancer surgery*?

### Participants

In this prospective part of the study 40 consecutive patients operated per centre (n = 160 (4 × 40)) will be included. Again we will use the same inclusion and exclusion criteria as in the primary implementation study.

### Variables and measurements

#### Policy cost analysis

The cost analysis will be performed from a societal perspective, using the Dutch guidelines for cost calculations in healthcare [26]. Policy costs represent both the programme and implementation costs [27]. The Short Stay Programme in breast cancer surgery related direct and indirect healthcare and non-healthcare costs will be measured and valued. We will use a bottom up micro costing method which identifies and measures the healthcare products per patient [26]. Costs will be expressed in 2012 Euros. Direct healthcare related resource use will be obtained using Case Record Forms. Direct costs outside healthcare and out of pockets costs will be collected through a cost diary filled out by patients. One day before surgery, patients will fill out the retrospective part of the cost book to determine the healthcare related costs for a period of two weeks before surgery. Patients will fill out the prospective part of the cost book during six weeks from the moment of discharge. This time horizon will cover most costs related to surgical treatment and is also used in the primary implementation study. To perform the cost calculations, the volumes of (healthcare) resource use are multiplied with the cost prices per unit of resource use. Cost prices from the primary study will be actualised and used in this study. Indirect costs are the productivity losses due to sick leave; these will be calculated using the friction costs method. A friction cost method confines productivity losses to the period needed to replace a sick worker.

Historical implementation costs of the Short Stay Programme in breast cancer surgery will be used in this study, as these costs have been made in the primary implementation study. The implementation costs were assessed as mean costs per patient.

#### Effectiveness

Effectiveness will be determined through a generic health-related quality of life (HRQoL) instrument. In this study the EuroQol (EQ-5D) will be used to calculate quality-adjusted life years (QALYs) [28]. The EQ-5D consists of five different dimensions (mobility, self-care, daily activities, pain/discomfort, depression/anxiety). Every dimension has three answer possibilities (no problems, some problems, severe problems), which can lead to 243 different health states. The health states as measured in this study will be used for utility score calculation, based on the UK tariff. The utility scores will be determined at four consecutive time points: one day before first surgery (at baseline), one day after first surgery, one week after first surgery and six weeks after first surgery.

### Data analyses

In this study the current incremental cost-effectiveness ratio (ICER) will be calculated for the Short Stay Programme in breast cancer surgery versus care as usual before primary implementation. For calculation of the ICER, mean incremental policy costs will be divided by the mean incremental QALYs. Multiple imputations will be used to replace missing values with plausible estimates. Bootstrapping will be performed to determine 95% confidence intervals around cost differences between the Short Stay Programme in breast cancer surgery and care as usual. Bootstrapping will also be used to quantify the uncertainty around the ICER and will be performed using Excel 2003 [29]. The results will be presented in an incremental cost-effectiveness plane in which the vertical axis will represent the incremental effects and the horizontal axis will show the incremental costs between the Short Stay Programme and care as usual before implementation. This will result in four quadrants: 1) southeast quadrant (SE) 2) northwest quadrant (NW) 3) southwest quadrant (SW) 4) northeast quadrant (NE). The cost-effectiveness acceptability curve will be calculated to present the probability of the Short Stay Programme being cost-effective for a range of ceiling ratios using Excel 2003. These analyses will be performed using SPSS 18.0 (SPSS Inc. Chicago, Illinois, USA). Sensitivity analyses will be performed to test the robustness of the results for changing several parameters, as well as subgroup analyses. In addition, current policy costs and effects of the Short Stay programme will be compared to policy costs and effects as calculated in the primary implementation study, and potential differences will be related to sustainability issues when applicable.

**Research question three**: *Which are the determinants of sustainability as perceived by the professionals of the Short Stay Programme in breast cancer surgery and the Enhanced Recovery After Surgery programme in colonic surgery*?

### Respondents

#### Professionals

Professionals within the participating hospitals will be interviewed. The first interview will be conducted with the responsible surgeon. Following this interview approximately 1-2 key persons in the actual care process per hospital will be selected. The number of interviews may differ per hospital based on the qualitative research process. Before the start of the interview, the interviewees will be formally informed about the relevance, expected duration, confidentiality of the personal data and the permission to audio tape the interviews.

#### External change agents

External change agents involved in the primary implementation strategies will also be invited for an interview to explore their perspective on sustainability in relation to the implementation processes and results.

### Variables and measurements

**Figure 1:**
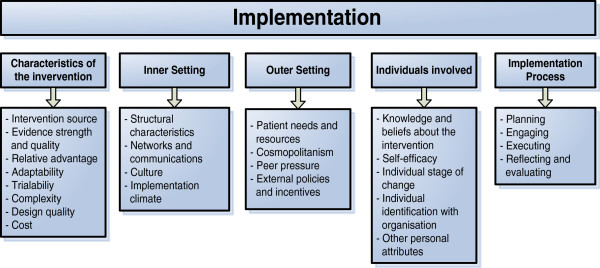
CFIR-Model: Damschroder et al, 2009.

The interview schedule (Additional file 3) for the semi structured interviews was developed based on a list of relevant topics inspired by the Consolidated Framework Implementation Research (CFIR) model, recently developed by Damschroder et al [30]. The CFIR model (Figure 1) is composed of 39 factors. These factors are organised into five constructs: the characteristics of the innovation, individuals involved, inner setting, outer setting and the implementation process [31-34]. Furthermore, the interview will be guided by information regarding current process and outcomes of care in comparison to historical results following primary implementation. The interviews will be held by one of the researchers (either SA or FG), who were not involved in the primary implementation process. First, after a short introduction the interviewees’ perception of sustainability in their hospital will be questioned. Second, hospital-specific information regarding current practice in comparison to historical results following primary implementation will be graphically displayed, and interviewees will be asked to reflect on these results. Third, interviewees will be questioned about the innovation itself, hospital culture, incentives and other factors possibly influencing sustainability. The interviews will be audio taped for documentation and analysis to guarantee transparency. Interviews will take about forty-five minutes. Immediately afterwards the researcher will make field notes. Summaries of the main findings per transcript will be sent to the particular hospitals to perform a member check.

### Data analysis

The interviews will be transcribed and independently coded by both researchers (SA and FG). Differences in coding will be discussed until consensus is reached. Research software NVivo will be used to analyse the data and progress in coding and analysis will be discussed in the research team.

## Discussion

This paper describes the protocol of a mixed methods observational study to gain more insight into the concept of sustainability and its determinants. Two healthcare innovations were implemented in a selection of Dutch hospitals three to five years ago, using different implementation strategies. A Short Stay Programme was implemented in breast cancer surgery in 4 early adopter hospitals by means of a hospital tailored implementation strategy, and the Enhanced Recovery After Surgery programme in colonic surgery was implemented in 33 hospitals by means of a generic implementation strategy. As a result of these efforts, both cases showed improved short-term results in terms of hospital length of stay. Currently, it is unclear to what extent these innovative programmes and their results have been sustained. In the current study, the extent of consolidation of the implemented programmes will be assessed in fourteen hospitals in the Netherlands; four hospitals will participate in the breast cancer surgery case and ten hospitals will participate in the colonic surgery case.

### Strengths and limitations of the study

This study will specifically and systematically address the sustainability of two healthcare innovations three to five years following two different implementation strategies. Regarding the breast cancer case, research on sustainability is not limited to long term effects of the implementation strategy, but also comprises policy cost-effectiveness. In the primary study, a policy cost effectiveness analysis on the Short Stay Programme was performed. As a consequence of e.g. changes in programme elements or case-mix, the current cost-effectiveness may be different. A recalculation of the cost-effectiveness will be conducted which will partly be based on the detailed cost- and effectiveness data collected during the primary study.

Another strength is the possibility to explore perceived determinants of sustainability from two different implementation strategies. Both programmes have been implemented in a multidisciplinary hospital setting. By studying these two different programmes, a large pool of medical specialists as well as other professionals can be approached. As such, this study will offer a multi-disciplinary perspective on sustainability and its determinants. We have chosen for the Consolidated Framework Implementation Research (CFIR) model to examine the determinants and the concept of sustainability. This framework is based on several scientific theories and existing models and considers a wide scope of possible determinants of sustainability.

In this study a mixed method approach will be applied, combining quantitative data with qualitative data. In the quantitative analyses we will examine whether the implementation results have been sustained, while in the qualitative analyses we will examine the professionals view on possible determinants of sustainability. The determinants of sustainability will be explored by means of semi-structured interviews with stakeholders of the healthcare processes and external change agents. This study is not aimed to analyse independent determinants in a quantitative manner by means of a multivariate regression model because we currently lack well-defined hypotheses on what are the most important determinants of sustainability. In this study we therefore also chose for qualitative methods.

Furthermore, the outcome and care process data will be extracted from patient files. The practical advantage is that the participating professionals are not claimed for intensive data collection by means of completing case record forms (CRFs). Also, by auditing the files of the last 40 breast cancer patients operated, and of the last 30 colonic surgery patients, a Hawthorne effect will be prevented. This is the phenomenon that a team is improving its performance due to awareness of being monitored.

This study has also some limitations. The generalisability of our quantitative results regarding sustainability will be limited due to the hospital selection. An analysis of sustainability of the ERAS programme in colonic surgery including all 33 participating implementation hospitals would have been optimal. Although the ten hospitals were selected based on carefully chosen criteria, it cannot be ruled out that some of the other hospitals that initially were not successful in achieving the ERAS goals, continued their activities and ultimately implemented the protocol successfully. The evaluation of the effects of the Short Stay Programme in breast cancer surgery after implementation will be conducted among the same hospitals as during the primary study. However, these early adopter hospitals may not fully represent Dutch practice regarding breast cancer surgery.

The analysis of sustainability is based on extracting data from existing data files. Although a Hawthorne effect is thus prevented, a potential limitation is that some data of interest for this study may not be properly recorded.

Not much is known about long term effects of implementation strategies and the determinants of sustainability. In this study, we perform an analysis on sustainability and we will try to shed light on potentially relevant attributes of sustainable change. We will build on previous work and aim to expand the knowledge regarding sustainability and its determinants. The results of this study will be relevant for researchers, implementation experts, healthcare practitioners and policy makers when making decisions about implementation and sustainability resource allocation.

## Competing interests

The authors declare that they have no conflicting interests.

## Authors’ contributions

SMCA is a health scientist, main investigator and PhD student. FG is a medical doctor, also main investigator and PhD student. MVM is the principal investigator and applicant of the proposed research. TvdW is co-applicant of this project. CDD and JMCM are the daily coordinators of the SUSHI-study. All authors read and approved the final manuscript.

## Pre-publication history

The pre-publication history for this paper can be accessed here:

http://www.biomedcentral.com/1472-6963/12/423/prepub

## Supplementary Material

Additional file 1 Short Stay Programme in breast cancer surgery. Click here for file

Additional file 2 Enhanced Recovery After Surgery programme in colonic surgery. Click here for file

Additional file 3 Interview themes for sustainability. Click here for file
