# Supplementary material for: Sustainability of healthcare innovations (SUSHI): long term effects of two implemented surgical care programmes (protocol)
Source: BMC Health Serv Res. 2012 Nov 23;12:423. doi: 10.1186/1472-6963-12-423 (PMC3545846; doi:10.1186/1472-6963-12-423)
Supplement: Additional file 1 — Short Stay Programme in breast cancer surgery. [file 1472-6963-12-423-S1.doc]

***Additional file 1. Short Stay Programme in breast cancer surgery***

Short Stay Programme in breast cancer surgery, ***study 2004-2007: Costs and effects of implementation of a short admission programme following breast cancer surgery in the Netherlands (M. de Kok, Maastricht University)***

The implementation of an ultra short stay programme was conducted in 4 different early adopter hospitals: one non-training hospital, one small training hospital, one large training hospital and a university medical centre. The aim of this study was to publish data on the different effects between centres, on the implementation, on the implementation strategy and on the policy cost-effectiveness.

The implementation and evaluation were highly successful. As a result, the ultra short stay after breast cancer surgery increased from 45% to 82% after using a highly tailored implementation in the participating hospitals. Breast-conserving surgery, having children and being employed were identified factors associated with an increase probability of short-stay admission. Being aged over 64 years indicated a trend towards a decreased chance for short stay admission. Quality of care stayed at a comparable level after the hospital tailored implementation of the short stay programme. Patient inspired goals for more quality improvement concerned education on drains, prosthesis, exercises after surgery, survival rates, and waiting and process times. The implementation costs were €23,- per patient and the programme was less costly (€7454) than care as usual (€8409). Furthermore, the implemented programme was cost-effective from a policy point of view. The ZonMW financially supported this major implementation project.

Implementation strategy: tailored

The comprehensive care programme for breast cancer surgery in ultra short stay was developed by the Maastricht University Medical Centre. Key figures in this programme were the multidisciplinary team, the breast nurse and the patient. A project leader was appointed and four early adopters (an active group with high status within the target group) within the field of breast cancer surgery were recruited. The first step in the implementation process concerned the assessment of the guideline recommendations by consensus among experts, noted in a few key recommendations. A so-called diagnostic analysis was performed to assess the usual care, and to explore factors that impede or facilitate the bridging of the gap between usual care and the programme to be implemented. This diagnostic analysis was based on interviews, meetings and telephone and email conferences. Perceived barriers and facilitators were identified during outreach visits to the hospitals in the preparation phase, described on different levels: study guideline, care provider, patient, colleague, organisation and financial resources and reimbursement.

After this phase a hospital-specific strategy was applied to implement a Short Stay Programme in breast cancer surgery programme. A multi-faceted implementation strategy was used, providing insight, inducing change and acceptance and feedback to maintain changes. This strategy was based on several components: 1) every early adopter appointed a breast nurse fully available for coordination of the programme; 2) high-frequency outreach visits and study group meetings representing all disciplines involved in breast cancer care, provided the forum in which all steps for implementation were prepared, and issues were discussed and resolved; 3) the care process was measured through indicators mentioned in Case Record Forms that were scored within a hospital, followed by feedback on performance by the researchers. The duration of the implementation strategy was six months, followed by six months of measurements ‘after implementation’.
