# Supplementary material for: Sustainability of healthcare innovations (SUSHI): long term effects of two implemented surgical care programmes (protocol)
Source: BMC Health Serv Res. 2012 Nov 23;12:423. doi: 10.1186/1472-6963-12-423 (PMC3545846; doi:10.1186/1472-6963-12-423)
Supplement: Additional file 2 — Enhanced Recovery After Surgery programme in colonic surgery. [file 1472-6963-12-423-S2.doc]

***Additional file 2. Enhanced Recovery After Surgery programme in colonic surgery***

ERAS programme in colonic surgery ***2005-2009: Implementation of an ‘Enhanced Recovery After Surgery’ programme in colonic surgery in thirty-three hospitals in the Netherlands using ‘The Breakthrough Series’ (J. Maessen, F. Gillissen, Maastricht University)***

The Enhanced Recovery After Surgery (ERAS) programme, describing eighteen elements of perioperative care, was developed to enhance functional recovery after surgery and to reduce the hospital length of stay (LOS) after colonic surgery. The ERAS programme was implemented under the guidance of the Dutch Institute for Health Care Improvement (CBO), using ‘The Breakthrough Series’ in 33 hospitals in the Netherlands, 3 university hospitals, 14 teaching hospitals and 16 non-teaching hospitals. The Breakthrough Series, characterised by four cycles of Plan-Do-Study-Act, was used to implement the ERAS programme. The aim of the ERAS study was to standardise perioperative care in colonic surgery and thereby to reduce the length of stay after surgery.

As a result, the median LOS after colonic surgery reduced by 3 days, from 9 to 6 days after the implementation of the ERAS programme in the participating hospitals. Laparoscopy, female sex, epidural anaesthesia, postoperative use of magnesium oxide, early mobilisation and early cessation of IV fluids were identified factors associated with an increased probability of shorter LOS, whereas ASA III-IV was an identified factor associated with a decreased probability of shorter LOS. The implementation was successful, and a dramatic change in perioperative routines was achieved, abandoning nasogastric tubes, abandoning bowel preparation before surgery and starting early nutrition after surgery.

Implementation strategy: The Breakthrough Series

The Breakthrough Series is an effective way for implementing change in clinical practice. It involves a structured collaboration of hospitals who share the wish to attain a known best practice in a certain field based on the Plan-Do-Study-Act structure. At first, site-visits by the CBO to the participating hospitals were performed and local multidisciplinary teams were formed, being a surgeon, an anaesthesiologist, a nurse, a research manager and, if possible, a member of the hospital management. Following this step, a two day start-up meeting in which the essential parts of the ERAS protocol were discussed as well as the involved local implementation process for change took place. Following this start-up meeting, each hospital made an individual project-plan, based on the assessment of the standard of care from the baseline measurement. Three-monthly learning sessions were organised by the expert team in which the expert team discussed areas of interest, while hospitals shared their local experiences. This allowed rapid dissemination of effective implementation strategies. During these learning sessions in the second and third group of hospitals, participants of the first group presented their implementation process. In between the learning sessions no contact was possible with the expert panel. Further site-visits in between these learning sessions by the CBO were aimed at supporting the local process of change (adaptation of the ERAS protocol to the local situation; dissemination to all parties within the hospital involved in perioperative care; planning, execution and evaluation of change). After one year, the implementation process was finished.
