# Supplementary material for: Sustainability of healthcare innovations (SUSHI): long term effects of two implemented surgical care programmes (protocol)
Source: BMC Health Serv Res. 2012 Nov 23;12:423. doi: 10.1186/1472-6963-12-423 (PMC3545846; doi:10.1186/1472-6963-12-423)
Supplement: Additional file 3 — Interview themes for sustainability. [file 1472-6963-12-423-S3.doc]

| What is your vision on the current results?  - Factors  Who was involved during the implementation process?  To what extent did these roles change?   - Leader   Are there any changes made in the programme?  - Protocol adherence  Are there any structural changes and activities executed after the implementation? (costs)  - Other investments  - Strategy  - Monitoring  Is there regular communication and feedback between involved professionals?  - Networks and structure  Is there an innovative culture within the hospital?  Are all programme elements understood by the involved professionals?  Were there changes on patient level?  - Patient needs  - Case mix  Do external policy and trends have an influence?  - Transparency performance indicators  What is the influence of other hospitals?  - Peer pressure  - Communication  Which multidisciplinary team members are involved?  What is the level of efficacy of the team members?  What are their support and beliefs about the programme?  What is the level of knowledge and training? |
| --- |

**Additional file 3. Interview themes for sustainability**
